# Bronchitis and Broncho-Pneumonia in Children

**Published:** 1906-03

**Authors:** William A. Wood

**Affiliations:** Gallatin, Mo.


For Texas Medical Journal.

### Bronchitis and Broncho-Pneumonia in Children.

BY WILLIAM A. WOOD, M. D., GALLATIN, MO.

Among the diseases of children at this season of the year none is more common or fatal than bronchitis and pneumonia. If anything we can say on this subject shall lead to a more careful study of these grave diseases in young children, our labor will not be in vain.

In dealing with this class of patients very many difficulties confront the physician. The infant can give him no information, and the child who is old enough to answer his questions intelligently very often refuses to come to his aid, hence subjective symptoms are not available in reaching a diagnosis. The thermometer is an unreliable guide for the reason that in children the temperature fluctuates. It rises suddenly and falls just as suddenly without any ascertainable cause. The pulse is also unsafe as a guide in disease, because the physician has no means of knowing what it is in normal health. Under the influence of nervous excitement, anger or fright, both the temperature and pulse are changeable and uncertain. When we add to these incidental influences the disturbing phenomena of disease, we begin to realize the embarrassment of the medical adviser.

The physician who is a close observer is, however, not without resources. He can get very much of the information he needs from the mother or nurse of the child and a patient study of the objective symptoms. There is a revelation in the cry of the child. The cry of pain is different from the cry of anger, or of hunger, or of exhaustion. He must note this difference. In pneumonia the cry is suppressed in consequence of its interference with respiration. The peculiarity of a cough must be studied. In ordinary bronchitis it does not cause pain. In pneumonia it is accompanied by more or less pain, which is plainly depicted on the face of the child. Physiognomy also teaches its lesson. Conditions of the countenance reveal the nature of disease. In sleep the face of a healthy child expresses repose. In pneumonia there is always a movement of the nostrils, indicative of difficult respiration. A chewing motion of the mouth denotes gastro-intestinal disturbance. It is said that the upper third of the face is changed in brain affections, the middle third in diseases of the chest, and the lower third in abdominal lesions. Contractions of the brows show pain in the head, and drawing of the upper lip, pain in the abdomen. A waxy color of the face indicates kidney disease, and a flush on the cheeks, inflammation of the lungs or pleura.

As broncho-pneumonia is secondary to bronchitis, it is not an easy matter to determine exactly when it begins, for its symptoms are often very obscure, and for this reason it is important to study all the objective symptoms which may aid in reaching a correct diagnosis.

Bronchitis is a very common disease in infancy and childhood. It is variable in extent and intensity. It begins with cough, hoarseness, difficulty of respiration and febrile excitement. There may be also soreness of the throat, coryza, sneezing, and a watery condition of the eyes. It may be ushered in with a chill or a chilliness with languor, exhaustion and drowsiness, followed later by more or less fever. The pulse becomes frequent, with a rise in the temperature. The cough may be slight at first, but increases in proportion to the extent and intensity of the inflammation. When it is frequent and severe it will be accompanied by more or less pain or soreness at the base of the sternum, but the face does not express the same degree of suffering as in the cough of pneumonia. The respiration in mild cases is but little accelerated, but in severe cases it is short, difficult and oppressed, and is attended by a wheezing or rattling sound, heard first in the throat, but subsequently over the whole of the chest.

The physical signs of acute bronchitis in very young subjects are a combination of mucous and sibilant rhonchi. In older children these sounds are more marked, especially the mucous rhonchi. When the inflammation extends to the more minute ramifications of the bronchi the general symptoms are correspondingly aggravated. We have now the capillary bronchitis of the older writers, which is exceedingly dangerous, generally terminating in death, sometimes in a few hours.

Every case of bronchial catarrh should be regarded as the beginning of pneumonia. In the commencement of an attack of bronchitis a small dose of calomel and Dover's powder, followed with castor oil, or salines, will be of service. Quinine now in small doses should be given at short intervals for about two days. Alternated with this, the following may be given:

℞ Vin ipecac ............................ʒj.

Syrup scilla ..........................ʒss.

Syrup senega ........................ ʒss.

Tinct. hyoscyamus ..................... ʒj.

Cord. ol. morrhuæ-comp. (Hagee) ........ ℥ii.

M. Sig.: Take from one-half to one teaspoonful, according to age.

The child should be kept warm with flannel next to the skin; should remain in bed, with the room at a uniform temperature of not less than 65° F., and should be given mucilaginous drinks or barley water with such nourishment as will best meet the wants of the system. Fomentations or hot cloths applied to the chest will often be of service.

If there is much prostration the following prescription may be given:

℞ Spts. ammon. arom. .................... ʒj.

Syrup senega ......................... ʒj.

Tinct. scilla .......................... ʒss.

Syr. prun. virg. ....................... ℥ij.

M. Sig.: Take from one-half to a teaspoonful every two hours.

In children of eight years and upward, the muriate of ammonia in small doses may be substituted for the spirits ammonia aromatic.

In case the inflammation has extended to the lung substance and broncho-pneumonia is developed, the chest should be enveloped in a jacket poultice of linseed meal covered with oiled silk, and if there is high fever give as follows:

℞ Potassii citratis ....................... ʒj.

Spts. ammonia arom. ................ gtt. xv.

Spts. ether nit. ......................... ʒss.

Liq. ammon. acet. .................... ʒiij.

Glycerin ............................. ℥ss.

M. Sig.: Give one-half to one teaspoonful every two hours.

If the cough is distressing, give the following:

℞ Vini antimon ......................... ʒss.

Spts. ether nit. ......................... ʒij.

Tinct. opii. camp. ...................... ʒij.

Cord. ol. morrhuæ comp. (Hagee) ....... ℥iss.

M. Sig.: From one-half to one teaspoonful every two hours.

Of course, symptoms as they arise must be met. It may be necessary to resort to aconite or digitalis or alcoholic stimulants, but the above is a general outline of the treatment in these cases.

